# Monaural Sound Localization Based on Reflective Structure and Homomorphic Deconvolution

**DOI:** 10.3390/s17102189

**Published:** 2017-09-23

**Authors:** Yeonseok Park, Anthony Choi, Keonwook Kim

**Affiliations:** 1 Division of Electronics & Electrical Engineering, Dongguk University-Seoul, Seoul 04620, Korea; dustjrdk@dongguk.edu; 2 Department of Electrical & Computer Engineering, Mercer University, 1501 Mercer University Drive, Macon, GA 31207, USA; choi_ta@mercer.edu

**Keywords:** sound localization, angle of arrival, monaural localization, acoustic reflection, time delay, homomorphic deconvolution, cepstrum, single microphone, far-field, 3D printer

## Abstract

The asymmetric structure around the receiver provides a particular time delay for the specific incoming propagation. This paper designs a monaural sound localization system based on the reflective structure around the microphone. The reflective plates are placed to present the direction-wise time delay, which is naturally processed by convolutional operation with a sound source. The received signal is separated for estimating the dominant time delay by using homomorphic deconvolution, which utilizes the real cepstrum and inverse cepstrum sequentially to derive the propagation response’s autocorrelation. Once the localization system accurately estimates the information, the time delay model computes the corresponding reflection for localization. Because of the structure limitation, two stages of the localization process perform the estimation procedure as range and angle. The software toolchain from propagation physics and algorithm simulation realizes the optimal 3D-printed structure. The acoustic experiments in the anechoic chamber denote that 79.0% of the study range data from the isotropic signal is properly detected by the response value, and 87.5% of the specific direction data from the study range signal is properly estimated by the response time. The product of both rates shows the overall hit rate to be 69.1%.

## 1. Introduction

Humans localize sound sources in three-dimensional (3D) space by using the binaural correlation and structure profile. The unique shapes of the head and pinna modify the propagated sound properties in the magnitude, phase and spectrum. The horizontal plane over the human head provides the binaural sound localization environment due to both ear positions. In an asymmetric manner, the direct and indirect distances from the source to the ears deliver clues for estimating the angle of arrival (AoA) in the plane. However, the median plane provides the symmetric space to the ears; therefore, the vertical position variation cannot be recognized by the simple correlation information between the ears. The pinna shape presents the acoustic variation of multiple reflections in the structure to identify the AoA over the median plane. Numerous papers have described the role of the pinna for median plane sound localization comprehensively [1,2,3,4,5,6,7,8,9,10,11]. Especially, Batteau [11] suggested the Laplace transform-based parametric model to understand the acoustic propagation over the pinna structure for extension to reverberation and other facets of human hearing. Further exploration of the pinna and head-related transfer function can be found by the recent papers [12,13]. Sound localization based on a single receiver, known as monaural localization (ML), is inspired by median-plane localization.

ML cannot be realized in the isotropic and far-field condition. The structure around the receiver should be placed for particular modification over the propagation path similar to the pinna structure. The receiver identifies the variation to estimate the AoA in the ML system. The asymmetric structure maximizes the acoustic property difference for individual localization angles. Numerous investigations have been conducted for a structure-related localization system and are summarized below. The designed analog circuit estimated the time difference between the direct and indirect propagation for ML [14]. The binaural system was extended for an extra dimension by using the pinna-like reflector and corresponding estimation algorithm [15,16,17,18,19]. The various structures around the microphone were explored to improve the directivity pattern of the head-related transfer function [20]. Based on the hidden Markov model and signal moment, the machine learning is proposed to approach the monaural sound localization with an artificial pinna [21]. The actively deformable pinna system is proposed and analyzed for sound localization in the application of a mobile robot [22]. From the artificial pinna, the sound source elevation is estimated by using the propagation transfer function and the neural network classifier [23]. The characteristics of indoor speech propagation were utilized for non-structural ML within a limited situation [24,25,26]. The parabolic structure with cepstral speech parameters was explored for position-dependent indoor ML [27]. A hybrid ML system based on the audio-visual method with cepstral parameters was proposed by Friedland et al. [28].

**Figure 1:**
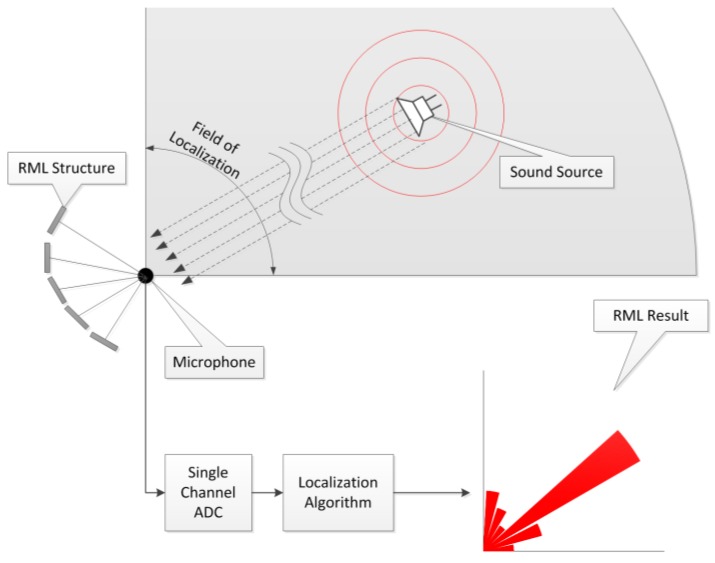
Functional diagram of the reflective monaural localization (RML) system.

This paper proposes a novel reflective monaural localization (RML) system for the far-field condition with the structure around the receiver. The structure provides the distinctive reflection times for individual angles to implant the direction information over the sound propagation. The received single-channel information is decoded to estimate the induced time delay by using homomorphic deconvolution (HD). The estimated delay is linearly mapped to the corresponding AoA in the final stage. Figure 1 shows the overall functional diagram. The incoming signal is discretized by the analog-to-digital converter (ADC), and the outcomes in terms of the likelihood of each direction are computed by the discrete process of the RML algorithm. A higher value represents an elevated possibility of an AoA. The designed structure should have at least one open face to the receiver and source to generate the proper reflections; therefore, the field of localization is limited to a certain range.

Along with the structure design, the time delay produced by the reflection is computed by the HD algorithm, which is established through the homomorphic system [29,30]. The HD removes or alters one of the components of a convolutional operation by using the homomorphic property. The HD is used for a variety of areas for single- and multi-dimensional signals, such as image, audio and seismic [31,32,33,34,35,36]. This paper realizes the HD based on the real cepstrum [37,38,39], lifting and inverse cepstrum procedure to estimate the propagation information, which delivers the time difference between the signal arrivals at the receiver. The source signal is eliminated by the lifting procedure, and the derived propagation information corresponds to the incoming AoA. Note that lifting is the term used by the cepstral analysis for filtering, and the cepstrum indicates the real cepstrum in this paper.

This paper accomplishes the work proposed by the authors’ previous ML publications. The fundamental frequencies induced by the asymmetric horizontal pyramidal horns were arranged for the far-field ML system by utilizing cepstral parameters [40]. The small-profile near-field ML system was realized by the asymmetric vertical cylindrical pipes around a single microphone [41]. The RML system of this paper improves the overall size from the pyramidal horn structure method, as well as the working range from the cylindrical pipe structure method. Other localization works on the subject by the authors are also related to and expanded during the research, such as azimuthal movement detection based on binaural architecture [42] and a target localization algorithm over a distributed acoustic sensor network [43]. Observe that the RML experiments are performed and evaluated within an identical anechoic chamber [44] to that used in the previous works.

## 2. Methodology

**Figure 2:**
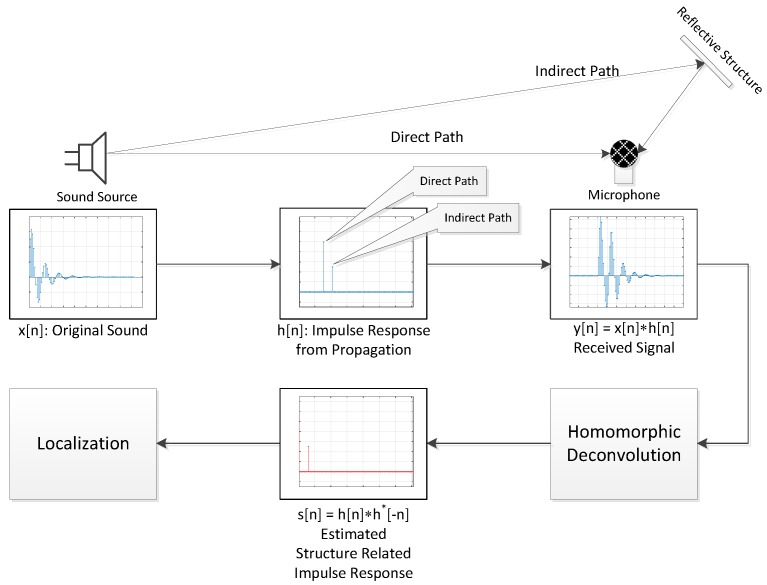
System architecture of the proposed RML system.

Multi-path propagation can be described as a linear time-invariant (LTI) system with convolutional operation. The direct and indirect arrivals at the receiver are represented by the corresponding temporal Kronecker delta functions (or delta functions) at the impulse response. The received output is developed by the convolution output between the source signal and the impulse response. Once the impulse response is derived from the received signal, the propagation path can be calculated by the response. The RML system is initiated from the fundamental idea that the reflective structure is placed and the deconvolution operation is performed to find the signal’s AoA. The careful structural layout provides the linear variation of the reflection time between the arrivals over the signal AoAs. The calculated impulse response contains arrival time information that can be translated into the AoA based on the reflection model. Figure 2 denotes the overall system architecture of the proposed RML system.

The source waveform x[n] propagates over the physical space characterized by the environment and structure. The impulse response h[n] of the space delivers the path and distance information of the propagation. The received information y[n] from the single receiver can be depicted by the convolution (shown as * operator) between the waveform x[n] and impulse response h[n]. Note that the impulse response h[n] cannot be measured directly without using the waveform x[n] information which is not available. The HD estimates h[n] in the form of the structure-related impulse response (SRIR) s[n], which is the autocorrelation of h[n]. The SRIR s[n] represents the time difference between the sound arrivals due to the nature of the autocorrelation shown as convolution (*) between h[n] and time reversed & conjugated $h^{*}\left[-n\right]$. The time information from the SRIR is translated into the AoA by applying the localization model that shows the relationship between the arrival angle and dominant reflection equivalently distinctive time difference. Note that the model is devised by the physical simulation and optimized by the acoustic experiments.

The equations below provide the HD computational procedure for the SRIR. The HD utilizes the real cepstrum, which applies the logarithm to the absolute discrete Fourier transform (DFT) outcome. If it is desired that the system obtains the original waveform or impulse response, the HD should employ the complex cepstrum that uses the logarithm for the raw DFT with extra complexity induced from phase unwrapping. The complex cepstrum preserves the phase information; therefore, no ambiguity can be experienced in the inverse procedure, such as maximum and minimum phase realization. The HD from the real cepstrum generates the autocorrelation of the impulse response, named the SRIR, because of the absolute operation on the DFT. The window function w[n] separates the SRIR from the received signal by utilizing the logarithm property. The index of the minimum value within the interesting range signifies the time difference between the first and second arrivals to the receiver. The corresponding time is computed by sampling the frequency parameter. The details of the HD derivations can be found in Appendix A. Note that the indexes for the time and cepstrum domains are equalized for convenience.

(1) $$\overset{\mathrm{Received}\;\mathrm{signal}}{\to }y\left[n\right]=x\left[n\right]*h\left[n\right]$$

(2) $$\overset{\mathrm{Real}\;\mathrm{cepstrum}}{\to }c_{y}\left[n\right]=\mathrm{IDFT}\{\log{\left|\mathrm{DFT}\left\{y\left[n\right]\right\}\right|}^{2}\}$$

(3) $$\overset{\mathrm{Window}}{\to }c_{u}\left[n\right]=c_{y}\left[n\right]w\left[n\right]$$

(4) $$\overset{\mathrm{Inverse}\;\mathrm{cepstrum}}{\to }s\left[n\right]=\mathrm{IDFT}\left\{e^{\mathrm{DFT}\{c_{u}\left[n\right]\}}\right\}\approx h\left[n\right]*h^{*}\left[-n\right]$$

(5) $$\overset{\mathrm{Find}\;\mathrm{minimum}\;\mathrm{location}}{\to }r=\underset{n=\left\{n_{\min}\leq n\leq n_{\max}\right\}\;}{\mathrm{argmin}}s\left[n\right]$$

(6) $$\overset{\mathrm{Compute}\;\mathrm{the}\;\mathrm{reflection}\;\mathrm{flight}\;\mathrm{time}}{\to }t_{d}=\frac{r}{f_{s}}$$

The equations below indicate the mathematical derivations for the HD algorithm on direct and indirect sound propagation. x[n] is the original waveform generated by the sound source, and $x\left[n-r_{1}\right]$ and $x\left[n-r_{2}\right]$ denote the first and second sound arrivals to the receiver, respectively. The second arrival is the reflected sound to the hard boundary; thus, the signal presents a phase reversal (π radian difference). –α represents the phase reversal with the reflection level. The HD output shows that $\delta \left[n-\left(r_{2}-r_{1}\right)\right]$ demonstrates the time difference between the arrivals with a minimum value. Observe that $\delta \left[n-\left(N-\left(r_{2}-r_{1}\right)\right)\right]$ is the shadow value created by the autocorrelation and DFT circular property. The HD outcome magnitude is independent of the signal amplitude and correlates to the reflection level α.

Received:

(7) $$y\left[n\right]=x\left[n-r_{1}\right]-\alpha x\left[n-r_{2}\right]\;\mathrm{where}\;r_{1}<r_{2}\;\mathrm{and}\;0<\alpha <1$$

Real cepstrum:

(8) $$\begin{array}{ll} \overset{\mathrm{DFT}}{\to }Y\left[k\right] & =X\left[k\right]e^{-j\frac{2\pi }{N}kr_{1}}-\alpha X\left[k\right]e^{-j\frac{2\pi }{N}kr_{2}}\\ & =X\left[k\right]\left(e^{-j\frac{2\pi }{N}kr_{1}}-\alpha e^{-j\frac{2\pi }{N}kr_{2}}\right) \end{array}$$

(9) $$\overset{\mathrm{Squared}\;\mathrm{Magnitude}}{\to }{\left|Y\left[k\right]\right|}^{2}=Y\left[k\right]Y^{*}\left[k\right]\;={\left|X\left[k\right]\right|}^{2}\left(e^{-j\frac{2\pi }{N}kr_{1}}-\alpha e^{-j\frac{2\pi }{N}kr_{2}}\right)\left(e^{j\frac{2\pi }{N}kr_{1}}-\alpha e^{j\frac{2\pi }{N}kr_{2}}\right)\;={\left|X\left[k\right]\right|}^{2}\left(1+\alpha^{2}-2\alpha \cos\left(\frac{2\pi }{N}k\left(r_{2}-r_{1}\right)\right)\right)$$

(10) $$\overset{\mathrm{Logarithm}}{\to }\log{\left|Y\left[k\right]\right|}^{2}\;=\log{\left|X\left[k\right]\right|}^{2}+\log\left(1+\alpha^{2}-2\alpha \cos\left(\frac{2\pi }{N}k\left(r_{2}-r_{1}\right)\right)\right)$$

(11) $$\overset{\mathrm{IDFT}}{\to }\mathrm{IDFT}\left\{\log{\left|Y\left[k\right]\right|}^{2}\right\}\;=\mathrm{IDFT}\{\log{\left|X\left[k\right]\right|}^{2}\}+\mathrm{IDFT}\left\{\log\left(1+\alpha^{2}-2\alpha \cos\left(\frac{2\pi }{N}k\left(r_{2}-r_{1}\right)\right)\right)\right\}$$

Window:

(12) $$\begin{array}{l} \overset{\mathrm{Window}}{\to }c_{u}\left[n\right]=\mathrm{IDFT}\left\{\log{\left|Y\left[k\right]\right|}^{2}\right\}w\left[n\right]\\ \approx \mathrm{IDFT}\left\{\log\left(1+\alpha^{2}-2\alpha \cos\left(\frac{2\pi }{N}k\left(r_{2}-r_{1}\right)\right)\right)\right\} \end{array}$$

Inverse cepstrum:

(13) $$\overset{\mathrm{DFT}}{\to }\mathrm{DFT}\{c_{u}\left[n\right]\}\approx \log\left(1+\alpha^{2}-2\alpha \cos\left(\frac{2\pi }{N}k\left(r_{2}-r_{1}\right)\right)\right)$$

(14) $$\overset{\mathrm{Exponential}}{\to }e^{\mathrm{DFT}\{c_{u}\left[n\right]\}}\approx 1+\alpha^{2}-2\alpha \cos\left(\frac{2\pi }{N}k\left(r_{2}-r_{1}\right)\right)$$

(15) $$\overset{\mathrm{IDFT}}{\to }\mathrm{IDFT}\left\{e^{\mathrm{DFT}\{c_{u}\left[n\right]\}}\right\}\;\approx \left(1+\alpha^{2}\right)\delta \left[n\right]-\alpha \delta \left[n-\left(r_{2}-r_{1}\right)\right]-\alpha \delta \left[n-\left(N-\left(r_{2}-r_{1}\right)\right)\right]$$

**Figure 3:**
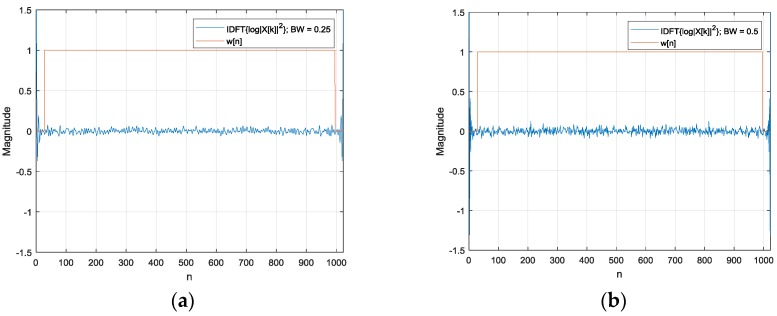

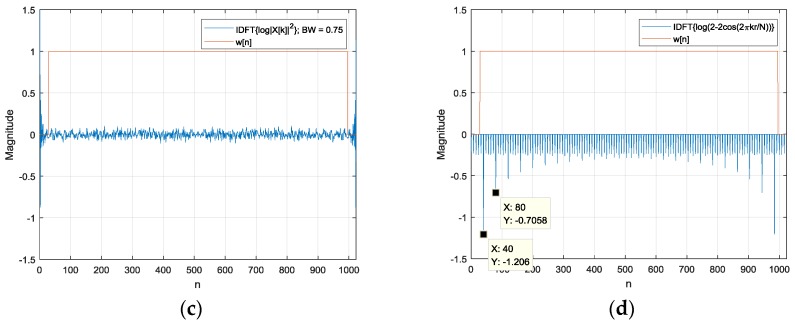
The signal portion $\mathrm{IDFT}\{\log{\left|X\left[k\right]\right|}^{2}\}$ for the (**a**) 0.25, (**b**) 0.5 and (**c**) 0.75 normalized frequency bandwidths and (**d**) the delay part $\log\left(2-2\cos\left(2\pi k\left(r_{2}-r_{1}\right)/N\right)\right)$ for 40 sample delays.

**Figure A1:**
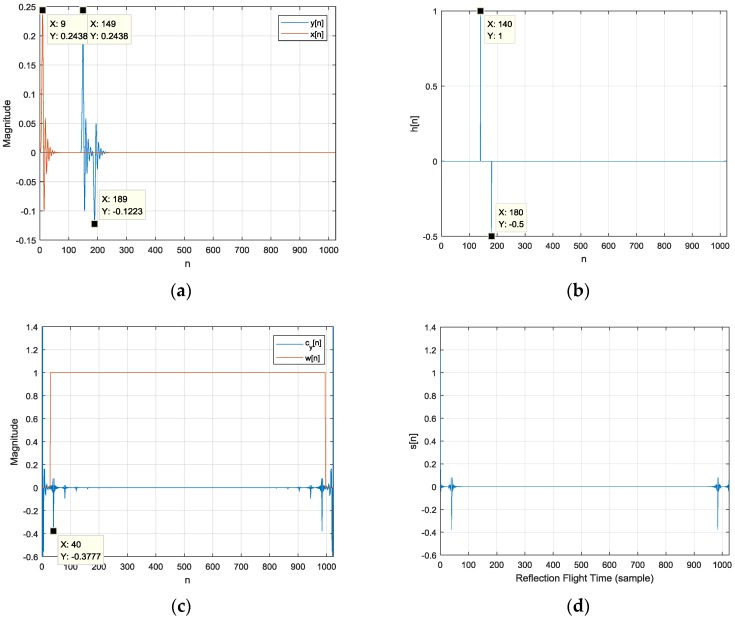
Homomorphic deconvolution for low-frequency signal based on the impulse response of the Butterworth filter (BW: 0.25). (**a**) Sound source *x*[*n*] and received signal *y*[*n*]; (**b**) impulse response of propagation; (**c**) real cepstrum output *c_y_*[*n*] and window function *w*[*n*]; and (**d**) the output of the homomorphic deconvolution as $\hat{s}\left[n\right]$.

$\mathrm{IDFT}\left\{\log{\left|Y\left[k\right]\right|}^{2}\right\}$ is the linear combination of the signal stand delay components in the above equations. The window function w[n] separates the delay part from the inverse DFT operation. An example is shown in Figure 3 with α=1 magnitude and $\left(r_{2}-r_{1}\right)=40$ samples. The signal is generated by the white noise with a 10th-order Butterworth low-pass filter for 0.25, 0.5 and 0.75 normalized frequency bandwidths. Note that the 1 normalized frequency indicates half of the sampling frequency. For the increased bandwidth, $\mathrm{IDFT}\{\log{\left|X\left[k\right]\right|}^{2}\}$ exhibits the amplified values in the overall range; however, a significant portion of the energy is concentrated on the edges. In contrast, the delay part $\log\left(2-2\cos\left(2\pi k\left(r_{2}-r_{1}\right)/N\right)\right)$ demonstrates the strong and damped values over the harmonics of the time difference. The delay part logarithm can be approximated by the Newton–Mercator series [45] for converging harmonics. The window w[n] including the interesting time delay can effectively extract the delay part for further processing. Note that the circular property of the real cepstrum requires the window in a circular manner. Figure A1 denotes the rest of the process for the HD and its outcome.

**Figure 4:**
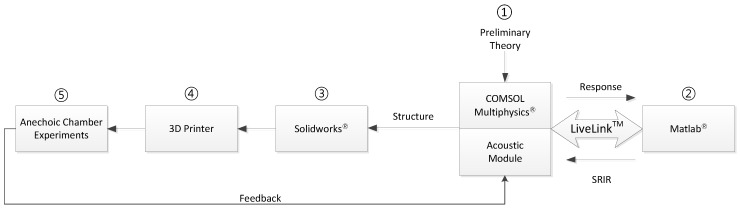
Workflow of the RML system development [41].

The structure of the RML consists of the combination of multiple plates to produce distinct time delays from individual directional signals. This paper comprehensively employs the procedure of modeling, simulation and experimentation to minimize design and experiment iterations. To create and evaluate the RML structure, Figure 4 presents the overall procedure, which follows an identical process to that of the previous study [41]. The initial structure is assessed by the COMSOL Multiphysics software to compute the signal propagation. MATLAB provides parametric variations and receives the temporal response to calculate the time delay from the HD algorithm. Procedures ① and ② in Figure 4 signify the simulation workflow to propose the structure shape. The candidate structure from the simulation is realized by the design program (SolidWorks) and a 3D printer for acoustic experiments in the anechoic chamber. The actual shape is placed in the anechoic chamber to analyze the received signal and examine the localization performance. Procedures ③, ④ and ⑤ in Figure 4 represent the experiment workflow for comprehensive structure feedback. Based on the acoustic performance, further modifications could be necessary for several iterations.

The acoustic experiments are executed and analyzed in an anechoic chamber that has been verified to exhibit partial conformance with ISO 3745 [46] for the 250 Hz–16 kHz one-third octave band in a free-field chamber and for the 1 kHz–16 kHz one-third octave band in a hemi-free-field chamber [44]. The RML system is evaluated with the free-field chamber mode, which contains fully-covered surfaces for all directions with acoustic wedges. Note that the RML structure indicates the physical structure, and the RML algorithm denotes the HD algorithm with the direction model from time delay to AoA. In addition, the RML system is the combination of the structure and the algorithm.

## 3. Structure Design and Simulation

**Figure 5:**
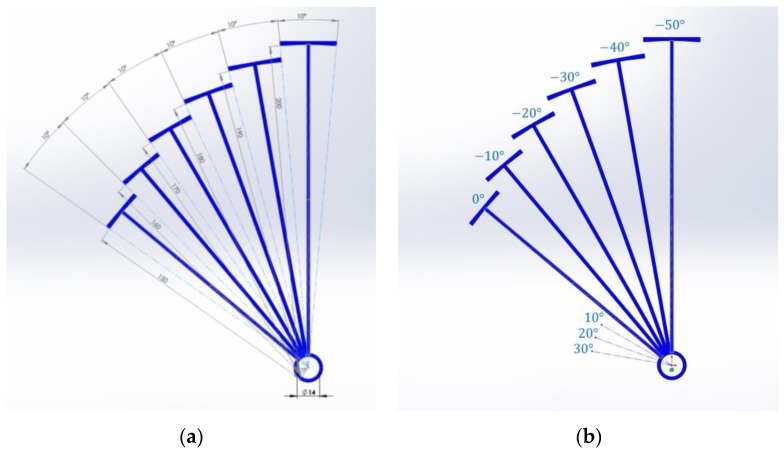
The designed RML structure for (**a**) dimensions in millimeters and (**b**) angles in degrees.

The designed RML structure consists of six plates, which involves 60° physical coverage, as shown in Figure 5. The plate is the arc of the circle with an individual radius to the center. The arc shows a fixed 10° central angle and a distance range from 150 mm to 200 mm in every 10 mm variation. The arc center also includes a concentric circle with a 7 mm radius for the receiver microphone. The azimuthal angle is measured over the RML structure plane from the leftmost arc center to the counterclockwise direction. Figure 5b demonstrates the corresponding angles for each arc. Note that the arc height is 100 mm, which is not illustrated in the figure below. To avoid acoustical distraction, the connection line from the arc to the center is developed as a low profile.

**Figure 6:**
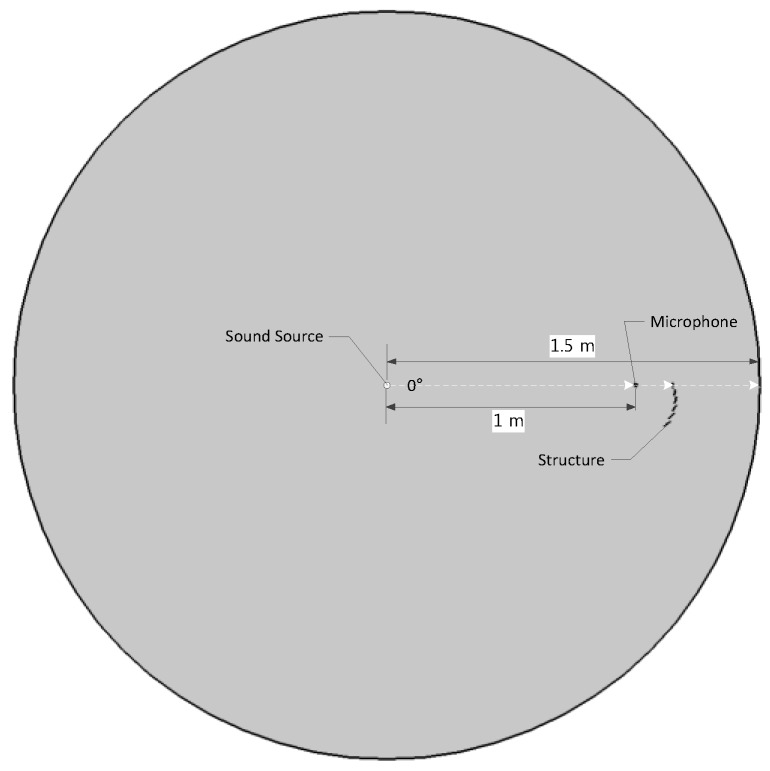
COMSOL simulation geometry for 0° angle of arrival (AoA). The RML structure is located in the air space (circle) with a perfectly absorbing boundary.

The proposed RML structure is evaluated by the COMSOL Multiphysics simulator for optimal parameters over multiple iterations. The simulation space is described by the two-dimensional (2D) space for a 1.5 m radius cylindrical free field as shown Figure 6. The principal interest of the analysis is acoustic propagation over the RML structure plane; thus, the 2D configuration provides sufficient information for parametric search. The circle boundary is established as a perfectly matched layer to represent an open and non-reflecting infinite domain for all wave types. The sound source is located at the circle center, and the receiver is placed 1 m away from the source in the eastbound direction for far-field provision. The RML structure is initiated from the microphone position, which is the 7 mm radius circle. The connection lines between the microphone and plates are not studied and shown in the simulation process. The rigid body of the structure is defined by the sound hard wall in the simulation for zero normal components of the velocity. The simulation space is filled with the air provided by the default simulator parameters. 

The sound source excites the air medium through a point Gaussian pulse in terms of amplitude, frequency bandwidth and pulse peak time. The complete parameters for the COMSOL simulation are described in Table A1 (Appendix B). With a 2 m^2^/s amplitude, 4 kHz bandwidth and 2.5 × 10^−6^ s peak time, the microphone receives the signals for −20°, −10°, 10° and 30°, as shown in the figure below. The first sound arrivals are identical to all AoAs; however, the second arrivals denote a phase reversal from the reflection with various magnitudes and delays. The minimum value at the second arrival corresponds to the maximum value at the first arrival due to the π radian phase difference. The times of the second impact minimum illustrate the inversely-proportional relationship with AoAs in a consistent manner. Therefore, the precise measurement of the time difference between the arrivals can be used to estimate the signal AoA.

**Figure 7:**
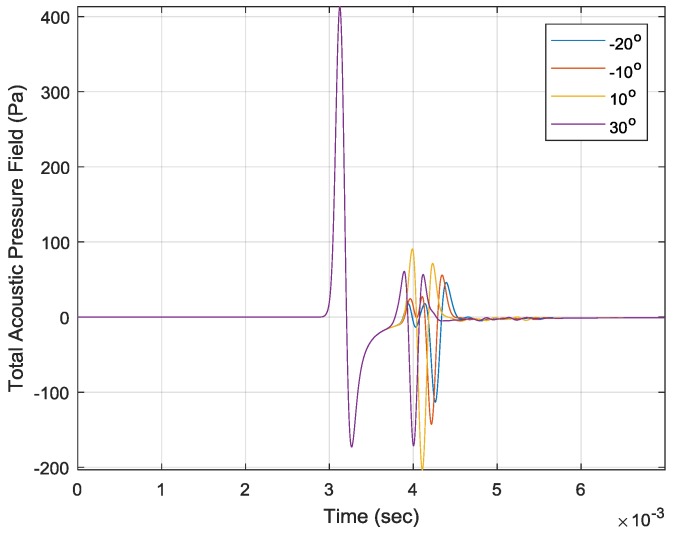
The received acoustic pressure field for various AoAs.

**Figure 8:**
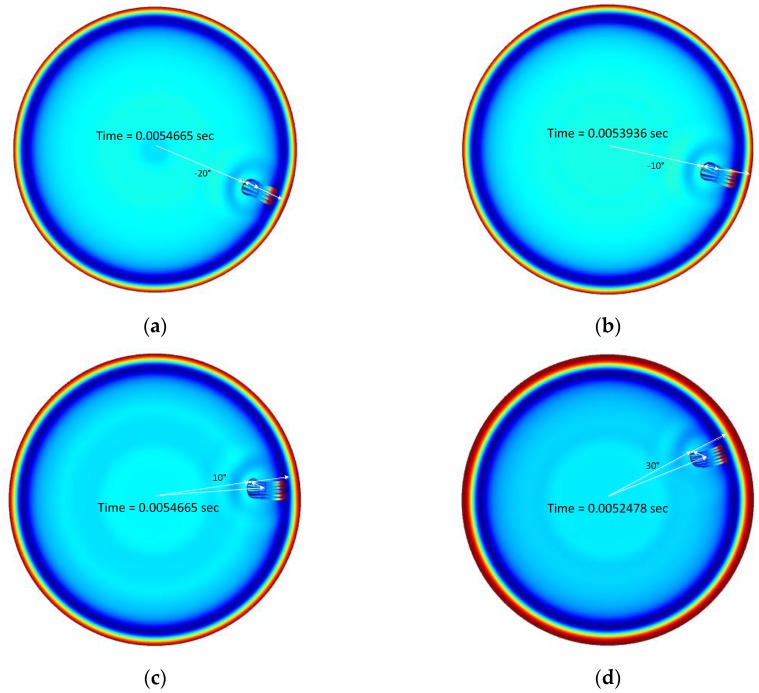
Total acoustic pressure field (surface plot) and local acceleration (streamline) for (**a**) −20°, (**b**) −10°, (**c**) 10° and (**d**) 30° AoA. Note that the white lines are intentionally inserted for visualizing the dominant propagation paths.

Because of the structure configuration shown in Figure 5b, the positive AoAs do not exhibit the normal reflection, which has a perpendicular incident and reflected angle to the plate. The propagation line does not connect the source, receiver and plate in the direct path; however, the reflection strength is relatively strong in Figure 7 for the 10° and 30° AoAs. The point Gaussian pulse collectively arrives in the direct path at approximately 3 ms; however, the reflected signals individually reach the receiver with a time difference between the 3.5 ms and 4.5 ms in Figure 7. Figure 8 denotes the surface plot for the total acoustic pressure field, the streamline for local acceleration and the white line for the dominant propagation path over −20°, −10°, 10° and 30° AoAs. The surface plot animations for successive time flow can be found with this *Sensors* paper on the MDPI website as Supplementary Materials. Figure 8 shows the simulation of the specific time indicated at the plot center. The Gaussian pulse is reached at the simulation boundary at the noted time, and a portion of the wave is reflected to other directions illustrated by the streamline. The normal reflections in Figure 8a,b are represented by the relatively straight streamline and propagation path. The non-perpendicular reflections in Figure 8c,d are depicted by the curved streamline and indirect path. The structure plates are properly designed to reflect the incoming signal toward the receiver for a non-normal incident over certain positive angles. Therefore, the strong second arrivals result from the non-perpendicular reflections.

**Figure 9:**
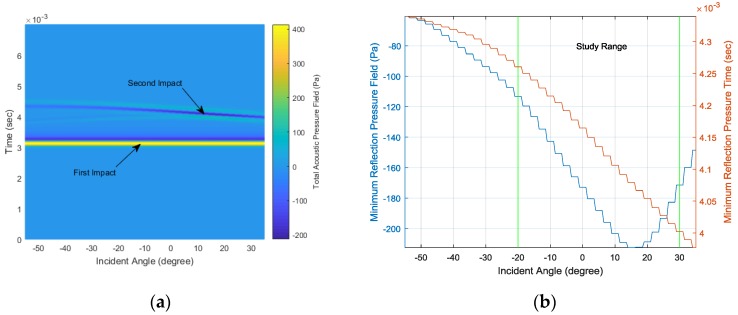
(**a**) Simulated received signals with various angles at a 2.5° step and (**b**) the second arrival magnitude (left y-axis) and time (right y-axis).

The LiveLink connection between the COMSOL simulator and MATLAB software provides a batch simulation process with continuous parameter variation. Figure 9 represents the simulation outcomes from the −55° to 35° AoA range in every 2.5° step. The surface plot in Figure 9a distinctly demonstrates the constant first impact at around 3 ms and the linear second impact in the time range. Figure 9b shows the second arrival magnitude in the left y-axis and corresponding time in the right y-axis. Note that the time is the absolute value and not the relative time between the first and second arrivals. The structure is devised to deliver the strong second arrival near 15° and the linear arrival time from −20° to 30° for optimal performance through a parametric search in the simulation. The designated scope is symbolized as the study range in Figure 9b. For a given AoA, the time delay induced by the structure is estimated by the HD algorithm and corresponds to the linear second arrival time shown in Figure 9b. Theoretically, the arrivals cannot be measured in the absolute time scale by using the single receiver; however, the time difference can be derived by using the HD algorithm.

**Figure 10:**
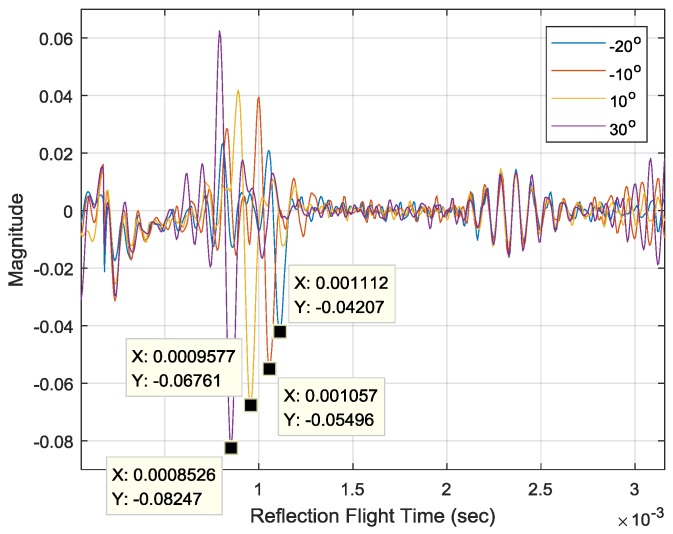
Simulated homomorphic deconvolution (HD) outputs for structure-related impulse response (SRIR).

The simulated Gaussian pulse response can be delivered to MATLAB for HD computation. The SRIRs from the HD algorithm are shown in Figure 10 for the −20°, −10°, 10° and 30° AoAs. Since the SRIR is the autocorrelation of the propagation response, the SRIR time represents the relative time scale equivalent to the time difference between the first and second impacts as the reflection flight time (RFT). The minimum value time in the SRIR indicates the highest likelihood for RFT that can be used for AoA computation. For increasing the AoA, the minimum time decreases at around 1 ms, as shown in the figure below with labels. The inversely-proportional relationship corresponds to the Gaussian pulse response plot in Figure 9b.

**Figure 11:**
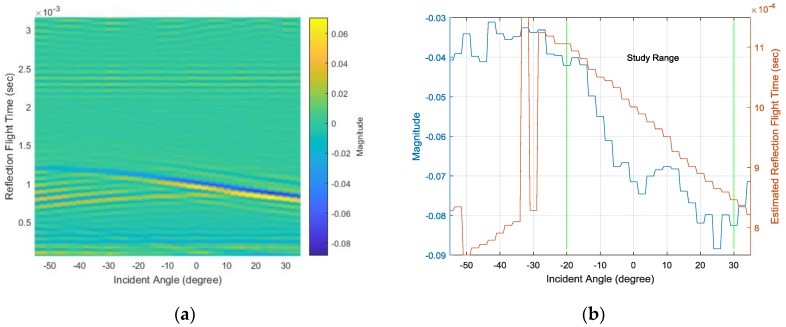
(**a**) Simulated SRIR with various angles at a 2.5° step and (**b**) the minimum magnitude (left y-axis) and reflection flight time (RFT) (right y-axis).

The simulated SRIR from the −55° to 35° AoA range is presented in Figure 11a with a 2.5° resolution. The minimum values denoted by the blue line gradually show the linear movement and intense depth within the −20°~30° AoA range. The corresponding value and time are illustrated in Figure 11b in the left and right y-axes, respectively. The magnitude in Figure 11b signifies the reflection magnitude –α in Equation (7) and is related to the Figure 9b magnitude. The reflection magnitude decreases overall and indicates the minimum value at around 25°. The RFT also linearly diminishes within the study range and can be convertible to the Figure 9b time by adding the first impact time. Due to the weak reflection, the range outside of the study range provides an elevated magnitude, as well as a fluctuating RFT. The consistency in the RFT is crucial information for determining the AoA with a simple linear model; therefore, the SRIR in the study range appropriately delivers clues for estimating the incident AoA.

This section presents the RML structure design and simulation results related to the HD algorithm to determine the SRIR capability. The optimal RML structure presented above is derived from the parametric search through an extensive simulation. The Gaussian pulse response clearly shows the first and second impacts induced by the propagation and reflection, respectively. The linear time difference between the arrivals is denoted by the SRIR from HD computation. Due to the phase reversal from the reflection, the minimum value and its time represent the reflection magnitude and flight time, correspondingly. The designed RML structure provides the linear RFT variation within the −20°~ 30° AoA range. Thus, the simple first-order model can convert the RFT to the incident AoA in the next section.

## 4. Results

**Figure 12:**
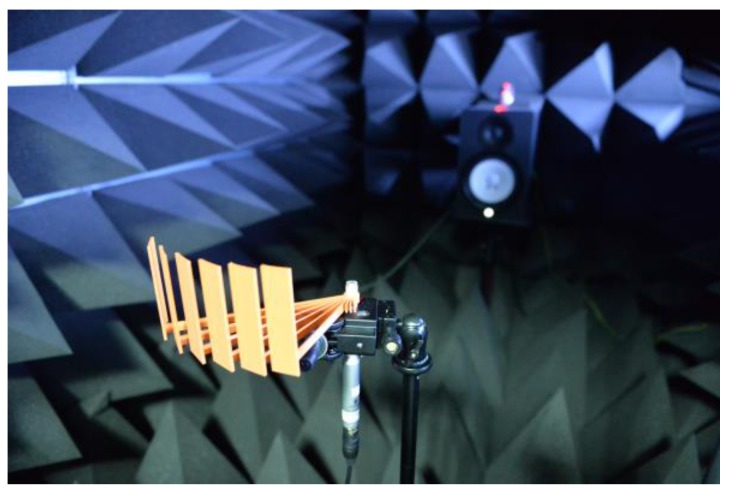
Acoustic experiment in the anechoic chamber with the 3D-printed RML structure.

The RML structure is realized by the 3D printer (Replicator 2, MakerBot, Brooklyn, NY, USA) based on the polylactic acid (PLA) filament and is illustrated in Figure 12. The acoustic experiments are performed and analyzed in an anechoic chamber, described in Section 2, with a free-field condition [44]. The structure is located in the direct-front direction 1.38 m away from the speaker. The small hole in the structure is loaded with the measurement microphone vertically, as shown in Figure 12. The MATLAB programming controls the microphone (ECM8000, Behringer, Tortola, British Virgin Islands), computer-connected audio device (Quad-Capture, Roland, Hamamatsu, Japan) and speaker (HS80M, Yamaha, Hamamatsu, Japan) simultaneously. The MATLAB system object with the audio stream input/output (ASIO) driver processes the real-time audio in terms of generation, reception and execution. Thus, the single-process iteration generates the SRIR outcome from the HD computation for the given structure and AoA.

**Figure 13:**
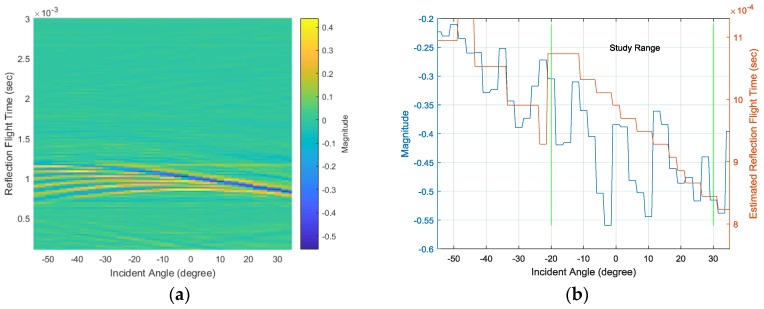
(**a**) SRIR experiment results for various angles at a 2.5° step and (**b**) the minimum magnitude (left y-axis) and RFT (right y-axis).

The white noise with a normal distribution generates the full uniform spectrum signal from the speaker. The data window length for the HD parameter is 1024 samples, and the 30 second data processing with a truncating-transition head and tail portion are ensemble-averaged to present the dominant SRIR. Note that the sampling frequency is 48 kHz for all experiments. The SRIR experiment results from the −55° to 35° AoA range are demonstrated in Figure 13a with a 2.5° resolution. Similar to the simulation results in Figure 11a, the blue line for the minimum values denotes the linear movement and intense depth within the −20°~30° AoA range. The corresponding value and time are plotted in Figure 13b in the left and right y-axes, respectively. The reflection magnitude decreases overall with higher variance than the simulation results shown in Figure 11b. The RFT consistently diminishes within the study range and harmonizes well with the simulation counterpart.

**Figure 14:**
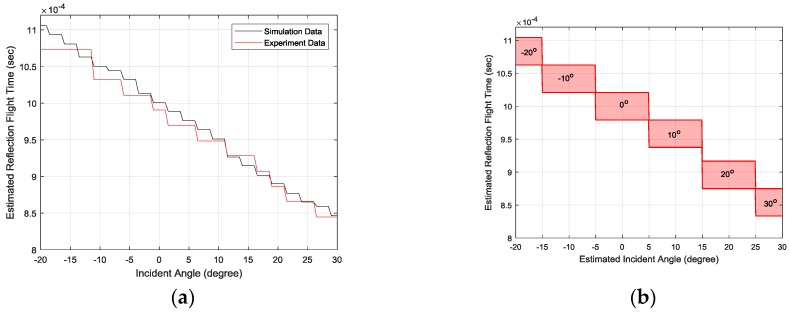
(**a**) The simulation and experimentation results for RFT from SRIR within the study range and (**b**) the discrete linear model for estimating AoA from RFT. Each red box represents the single 10-degree angle, such as −20° and −10°.

The consistent linearity of the RFT is important information for determining the RML system feasibility. Once the linearity is preserved, the individual RFT range corresponds to the specific AoA by using first-order arrangement. The simulation and experimentation results for the RFT are illustrated in Figure 14a. The experiment RFT outcome follows the simulation counterpart well in terms of linearity, as well as value. The AoA estimation model for the given experiment is derived in Figure 14b. The output AoAs are divided into six angles from −20° to 30° with a 10° resolution, and the corresponding RFT range is illustrated in the figure. For example, the RFT scope from 1.06 ms to 1.10 ms represents the −20° AoA. From 0.83 ms to1.10 ms, the six discrete RFT scopes are continuously distributed for six AoAs. Note that the RFT decision gap located between 10° and 20° does not influence the estimation performance because of the RFT distribution.

**Figure 15:**
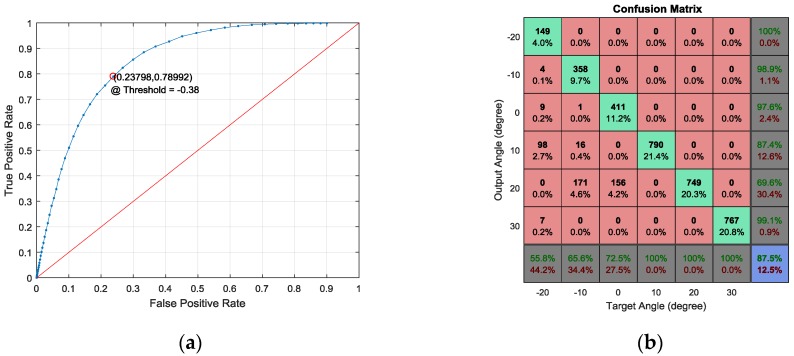
(**a**) The receiver operating characteristic (ROC) curve for intrinsic AoA range detection and (**b**) the confusion matrix for given discrete AoA.

The RML system is designed to estimate the limited field of localization from the −20° to 30° AoA range. Angles outside this range are discriminated by the reflection magnitude to exclude the given AoA from the localization process. The optimal threshold for the reflection magnitude is derived from the receiver operating characteristic (ROC) curve shown in Figure 15a. A total of 33,768 data frames (16,884 within and 16,884 outside range) are evaluated for the ROC curve, and the individual frames are evenly distributed over the designated AoA range. The single frame is equivalent to the 1024 sample data window. The true positive rate (TPR) is the ratio of the number of true positives to the number of positive conditions. The false positive rate (FPR) is derived by dividing the number of false positives by the number of negative conditions. A positive condition indicates the data frame from the study range, and a negative condition specifies a frame from outside of the range. As the decision threshold is changed from minimum to maximum, the TPR and FPR change the value in a complementary manner, as shown in Figure 15a. The area near the upper-left corner shows the best detection performance due to the perfect TPR and zero FPR of the corner; therefore, the decision threshold of −0.38, which is the nearest point to the corner, produces the highest statistical performance (78.99% TPR and 23.80% FPR).

Below the −0.38 reflection magnitude, the RML system considers the given signal as data from the localization field. Below the threshold, the RML algorithm performs the HD for the SRIR and finds the minimum value for the RFT over the AoA. According to the structure angular distance between the plates, the resolvable AoA is determined to be 10° for the study range. Thus, the acoustic experiment is executed for the AoA range from −20° to 30° with a 10° resolution. Figure 15b demonstrates the confusion matrix for the designated angles. The individual data frame for the angle is selected randomly without prior knowledge of the AoA; therefore, the number of frames for the dataset is distributed with variance. The total data frames for the confusion matrix is 3686. The number of data frames for −20°, −10°, −0°, 10°, 20° and 30° is 267, 546, 567, 790, 749 and 767, respectively. Note that data used in the confusion matrix are assumed to belong to the study range AoA. 

The AoA for each column vector of the matrix represents the target AoA (condition) in which the signal is incident. The AoA for each row vector indicates the output AoA (test outcome) that the RML system determines. The green and red rates on the last row signify the true-positive rate (hit rate) and false-negative rate (miss rate) for each AoA, respectively. In the bottom-right-hand corner, the overall values of the hit and miss rates are numerated. Except the bottom row and rightmost column, the confusion matrix elements denote the decision counts and overall percentile. The diagonal elements with a bright green color demonstrate the counts and percentile for the true-positive case. Observe that the percentile shows the ratio to total events. The performance for an individual AoA is represented in the last row as the hit rate.

As the AoA increases, the hit rate increases substantially (i.e., from 55.8% to 100%). The negative and zero angles that propagate the signal over the single direct line with an identical reflection path show the deteriorated hit rate performance. However, the positive angles that deliver the signal over the independent path for the propagation and reflection demonstrate the perfect hit rate. The −20°, −10° and 0° AoAs are dominantly misinterpreted for 10°, 20° and 20°, correspondingly. The structure around the major normal reflection plate creates early bounces that cause decision confusion. The more plates near the direct angle present a lower hit rate overall. For example, the −20° AoA has two plates on one side and three plates on the other. The 0° AoA has zero plates on one side and five plates on the other. Further plates on both sides show the deteriorated decision accuracy performance. The positive AoAs do not include the plate on the direct propagation line, which provides a straight connection between the source and receiver. The reflection is induced by the obliquely-located plates and distributed widely in direction to avoid AoA decision ambiguity. The focused reflection to the receiver provides the dominant temporal information for the RML algorithm. The overall hit rate for the AoA study range is 87.5% and 69.1% for all ranges (0.875 × 0.790) according to the ROC TPR 79.0%.

## 5. Conclusions

This paper presents a novel localization method for finding the arrival angle of far-field sound propagation with a single microphone. The reflection structure over the microphone produces direction-wise temporal variation, which can be estimated by the structure-related impulse response from homomorphic deconvolution. The structure consists of six vertical rectangular plates with a 10° angular (from 0° to negative angles) and a 1 cm radius (from 15 cm to increasing distance) difference. Depending on the incident angle, the reflection signal experiences a distinctive propagation path for a unique flight time. According to the simulation and experiment, the proposed structure provides the linear variation of the time difference between the first and second arrivals for consistent movement. The homomorphic deconvolution used in this paper utilizes the real cepstrum and inverse cepstrum sequentially to derive the spatial propagation response’s autocorrelation, named the structure-related impulse response. The reflection physics is represented by the minimum value and time at the response in the algorithm. The simulation and experiment demonstrate that the prominent value and linear time can be observed from the −20° to 30° scope; therefore, the angle span is specified as the study range. The acoustic experiments in the anechoic chamber denote that 79.0% of the study range data from the isotropic signal is properly detected by the response value, and 87.5% of the specific direction data from the study range signal is properly estimated by the response time. The product of both rates (0.875 × 0.790) show the overall hit rate to be 69.1%.

The novelty of this paper can be found in the structure, algorithm and their connection. The structure is devised from the extensive parametric search simulations for optimal reflections in magnitude and time. The conventional homomorphic deconvolution employs the complex cepstrum to estimate the input signal or the propagation response. This proposition includes the homomorphic deconvolution for the autocorrelation of the propagation response based on the real cepstrum. The mutual direction optimizations between the structure and algorithm are exercised for the increased hit rate and decreased miss rate, as well. The fine-grained simulation is also included in the design procedure along with the preliminary modeling of the acoustic reflection; thus, the localization accuracy is improved considerably for the far-field sound source. This article is part of the future research described in the previous paper [41] as the near-field monaural localization extension to the far-field system. Future work will offer structure and algorithm enhancement for the comprehensive field of localization. The selection potential in terms of acoustic structures and estimation algorithms is considerable. Together with the continuous structure for high-resolution localization, future work will include the utilization of various structure architectures. The algorithm will be devised as a mathematical model to represent the received information by its coefficients as a parametric method. The similarity between the consecutive datasets will be explored by temporal post-processing in a statistical manner. With all of the above, the development of three-dimensional monaural localization for the azimuthal and elevation directions is the final objective of this research.

## Supplementary Materials

The following are available online at www.mdpi.com/1424-8220/17/10/2189/s1: Video S1: The −20° incident angle simulation video, Video S2: The −10° incident angle simulation video, Video S3: The 10° incident angle simulation video, Video S4: The 30° incident angle simulation video.

## Abbreviations

The following abbreviations are used in this manuscript (listed by order of appearance in the text).

3D	Three-dimensional
AoA	Angle of arrival (AoAs, angles of arrival)
ML	Monaural localization
RML	Reflective monaural localization
HD	Homomorphic deconvolution
ADC	Analog-to-digital converter
LTI	Linear time-invariant
SRIR	Structure-related impulse response
DFT	Discrete Fourier transform
IDFT	Inverse discrete Fourier transform
2D	Two-dimensional
RFT	Reflection flight time
PLA	Polylactic acid
ASIO	Audio stream input/output
ROC	Receiver operating characteristic
TPR	True positive rate
FPR	False positive rate
	

## Appendix A

The equations below illustrate the homomorphic deconvolution procedure based on the real cepstrum. The received signal y[n] is propagated through the two cascade linear time-invariant systems as $h_{1}\left[n\right]$ and $h_{2}\left[n\right]$. The sound source is x[n]. In Equation (A9), $\hat{x}\left[n\right]+{\hat{x}}^{*}\left[-n\right]$ is represented by $c_{x}\left[n\right]$, etc. We assume that the window function w[n] can successfully separate $c_{h_{1}}\left[n\right]+c_{h_{2}}\left[n\right]$ from $c_{y}\left[n\right]$ in Equation (A10). The final output of the homomorphic deconvolution is the autocorrelation of $h_{1}\left[n\right]*h_{2}\left[n\right]$, as shown in Equation (A13).

(A1) $$y\left[n\right]=x\left[n\right]*h_{1}\left[n\right]*h_{2}\left[n\right]$$

(A2) $$\overset{\mathrm{DFT}}{\to }Y\left[k\right]=\overset{N-1}{\underset{n=0}{\sum }}y\left[n\right]e^{-j\frac{2\pi }{N}kn}=X\left[k\right]H_{1}\left[k\right]H_{2}\left[k\right]$$

(A3) $$\overset{\mathrm{Squared}\;\mathrm{Magnitude}}{\to }{\left|Y\left[k\right]\right|}^{2}={\left|X\left[k\right]\right|}^{2}{\left|H_{1}\left[k\right]\right|}^{2}{\left|H_{2}\left[k\right]\right|}^{2}$$

(A4) $$\overset{\mathrm{Logarithm}}{\to }2\log\left|Y\left[k\right]\right|=2\log\left(\left|X\left[k\right]\right|\left|H_{1}\left[k\right]\right|\left|H_{2}\left[k\right]\right|\right)\;\;=2\log\left|X\left[k\right]\right|+2\log\left|H_{1}\left[k\right]\right|+2\log\left|H_{2}\left[k\right]\right|$$

(A5) $$\overset{\mathrm{IDFT}}{\to }c_{y}\left[n\right]=\frac{2}{N}\overset{N-1}{\underset{n=0}{\sum }}\log\left|Y\left[k\right]\right|e^{j\frac{2\pi }{N}kn}$$

(A6) $$=\frac{2}{N}\overset{N-1}{\underset{n=0}{\sum }}\log\left|X\left[k\right]\right|e^{j\frac{2\pi }{N}kn}+\frac{2}{N}\overset{N-1}{\underset{n=0}{\sum }}\log\left|H_{1}\left[k\right]\right|e^{j\frac{2\pi }{N}kn}+\frac{2}{N}\overset{N-1}{\underset{n=0}{\sum }}\log\left|H_{2}\left[k\right]\right|e^{j\frac{2\pi }{N}kn}$$

(A7) $$=\frac{1}{N}\overset{N-1}{\underset{n=0}{\sum }}\log\left(X\left[k\right]X^{*}\left[k\right]\right)e^{j\frac{2\pi }{N}kn}+\frac{1}{N}\overset{N-1}{\underset{n=0}{\sum }}\log\left(H_{1}\left[k\right]H_{1}^{*}\left[k\right]\right)e^{j\frac{2\pi }{N}kn}\;\;+\frac{1}{N}\overset{N-1}{\underset{n=0}{\sum }}\log\left(H_{2}\left[k\right]H_{2}^{*}\left[k\right]\right)e^{j\frac{2\pi }{N}kn}$$

(A8) $$=\frac{1}{N}\overset{N-1}{\underset{n=0}{\sum }}\left(\log X\left[k\right]+\log X^{*}\left[k\right]\right)e^{j\frac{2\pi }{N}kn}+\frac{1}{N}\overset{N-1}{\underset{n=0}{\sum }}\left(\log H_{1}\left[k\right]+\log H_{1}^{*}\left[k\right]\right)e^{j\frac{2\pi }{N}kn}\;\;+\frac{1}{N}\overset{N-1}{\underset{n=0}{\sum }}\left(\log H_{2}\left[k\right]+\log H_{2}^{*}\left[k\right]\right)e^{j\frac{2\pi }{N}kn}$$

(A9) $$=\hat{x}\left[n\right]+{\hat{x}}^{*}\left[-n\right]+{\hat{h}}_{1}\left[n\right]+{\hat{h}}^{*}{}_{1}\left[-n\right]+{\hat{h}}_{2}\left[n\right]+{\hat{h}}^{*}{}_{2}\left[-n\right]\;\;=c_{y}\left[n\right]=c_{x}\left[n\right]+c_{h_{1}}\left[n\right]+c_{h_{2}}\left[n\right]$$

(A10) $$\overset{\mathrm{Window}}{\to }c_{u}\left[n\right]=c_{y}\left[n\right]w\left[n\right]\approx c_{h_{1}}\left[n\right]+c_{h_{2}}\left[n\right]\;\;=\frac{1}{N}\overset{N-1}{\underset{n=0}{\sum }}\log{\left|H_{1}\left[k\right]\right|}^{2}e^{j\frac{2\pi }{N}kn}+\frac{1}{N}\overset{N-1}{\underset{n=0}{\sum }}\log{\left|H_{2}\left[k\right]\right|}^{2}e^{j\frac{2\pi }{N}kn}$$

(A11) $$\overset{\mathrm{DFT}}{\to }U\left[k\right]=\overset{N-1}{\underset{n=0}{\sum }}c_{u}\left[n\right]e^{-j\frac{2\pi }{N}kn}\approx \log{\left|H_{1}\left[k\right]\right|}^{2}+\log{\left|H_{2}\left[k\right]\right|}^{2}$$

(A12) $$\overset{\mathrm{Exponential}}{\to }e^{\log{\left|H_{1}\left[k\right]\right|}^{2}+\log{\left|H_{2}\left[k\right]\right|}^{2}}=e^{\log{\left|H_{1}\left[k\right]\right|}^{2}}e^{\log{\left|H_{2}\left[k\right]\right|}^{2}}={\left|H_{1}\left[k\right]\right|}^{2}{\left|H_{2}\left[k\right]\right|}^{2}$$

(A13) $$\overset{\mathrm{IDFT}}{\to }\frac{1}{N}\overset{N-1}{\underset{n=0}{\sum }}{\left|H_{1}\left[k\right]\right|}^{2}{\left|H_{2}\left[k\right]\right|}^{2}e^{j\frac{2\pi }{N}kn}=\frac{1}{N}\overset{N-1}{\underset{n=0}{\sum }}H_{1}\left[k\right]H_{1}^{*}\left[k\right]H_{2}\left[k\right]H_{2}^{*}\left[k\right]e^{j\frac{2\pi }{N}kn}\;\;=h_{1}\left[n\right]*h_{1}^{*}\left[-n\right]*h_{2}\left[n\right]*h_{2}^{*}\left[-n\right]=\hat{s}\left[n\right]$$

The subsequent example demonstrates the homomorphic deconvolution for a low-frequency signal. The signal is derived from the impulse response of the Butterworth filter with the 0.25 normalized bandwidth. Figure A1a shows the sound source x[n] (red plot) and received signal y[n] (blue plot). Figure A1b represents the impulse response of the propagation as $h_{1}\left[n\right]*h_{2}\left[n\right]$. Figure A1c denotes the real cepstrum outcome $c_{y}\left[n\right]$ and window function w[n]. The significant power of x[n] is concentrated on the low index portion of the real cepstrum; therefore, the window function can separate the impulse response exhibited by the harmonics of the delta function. The final output $\hat{s}\left[n\right]$ of the algorithm is presented by Figure A1d as the autocorrelation of the impulse response. The difference between the first and second arrivals, 40 samples (180–140), is derived in the result.

## Appendix B

**Table A1:** COMSOL Multiphysics simulator parameters.

Name	Value	Description
f0	4000 Hz	Frequency Bandwidth ^1^
A	2 m^2^/s	Amplitude ^1^
t0	2.5 × 10^−6^ s	Peak Time ^1^
N	6	Mesh Elements/λ
C	343 m/s	Sound Speed
h_max	0.014292 m	Maximum Mesh Size
fs	4.8 × 10^5^ Hz	Sampling Frequency
CFL	0.05	Relationship between Mesh Size and Time-step
Delt	2.0833 × 10^−6^ s	Maximum Time-step

^1^ Gaussian pulse.
